# Recent developments in ionization techniques for single-cell mass spectrometry

**DOI:** 10.3389/fchem.2023.1293533

**Published:** 2023-12-07

**Authors:** Qingli Zeng, Meng-Chan Xia, Xinchi Yin, Simin Cheng, Zhichao Xue, Siyuan Tan, Xiaoyun Gong, Zihong Ye

**Affiliations:** ^1^ Zhejiang Provincial Key Laboratory of Biometrology and Inspection & Quarantine, College of Life Sciences, China Jiliang University, Hangzhou, China; ^2^ Technology Innovation Center of Mass Spectrometry for State Market Regulation, Center for Advanced Measurement Science, National Institute of Metrology, Beijing, China; ^3^ National Anti-Drug Laboratory Beijing Regional Center, Beijing, China

**Keywords:** single-cell analysis, mass spectrometry, ionization, nanoelectrospray ionization, laser desorption ionization, secondary ion mass spectrometry, inductively coupled plasma

## Abstract

The variation among individual cells plays a significant role in many biological functions. Single-cell analysis is advantageous for gaining insight into intricate biochemical mechanisms rarely accessible when studying tissues as a whole. However, measurement on a unicellular scale is still challenging due to unicellular complex composition, minute substance quantities, and considerable differences in compound concentrations. Mass spectrometry has recently gained extensive attention in unicellular analytical fields due to its exceptional sensitivity, throughput, and compound identification abilities. At present, single-cell mass spectrometry primarily concentrates on the enhancement of ionization methods. The principal ionization approaches encompass nanoelectrospray ionization (nano-ESI), laser desorption ionization (LDI), secondary ion mass spectrometry (SIMS), and inductively coupled plasma (ICP). This article summarizes the most recent advancements in ionization techniques and explores their potential directions within the field of single-cell mass spectrometry.

## Introduction

Cells are the foundational biological blocks that comprise every living entity. Due to the significant heterogeneity among cells, the analysis of individual organisms or biological tissues can hardly reflect the patterns of life activities at the level of individual cells (Wang et al., 2020). To understand the essence of microscopic life activities more profoundly and acquire a more comprehensive and precise understanding of cytological information, related research must be conducted on a unicellular scale (Zenobi, 2013; Xiong et al., 2016). Nevertheless, unicellular analysis has been constrained by the minimal size of cells, numerous types of substances, extremely low levels of compounds, and significant concentration differences between various compounds (Xiong et al., 2016; Yin et al., 2019). Therefore, in-depth research on single-cell analysis technologies has become extremely important.

**FIGURE 1:**
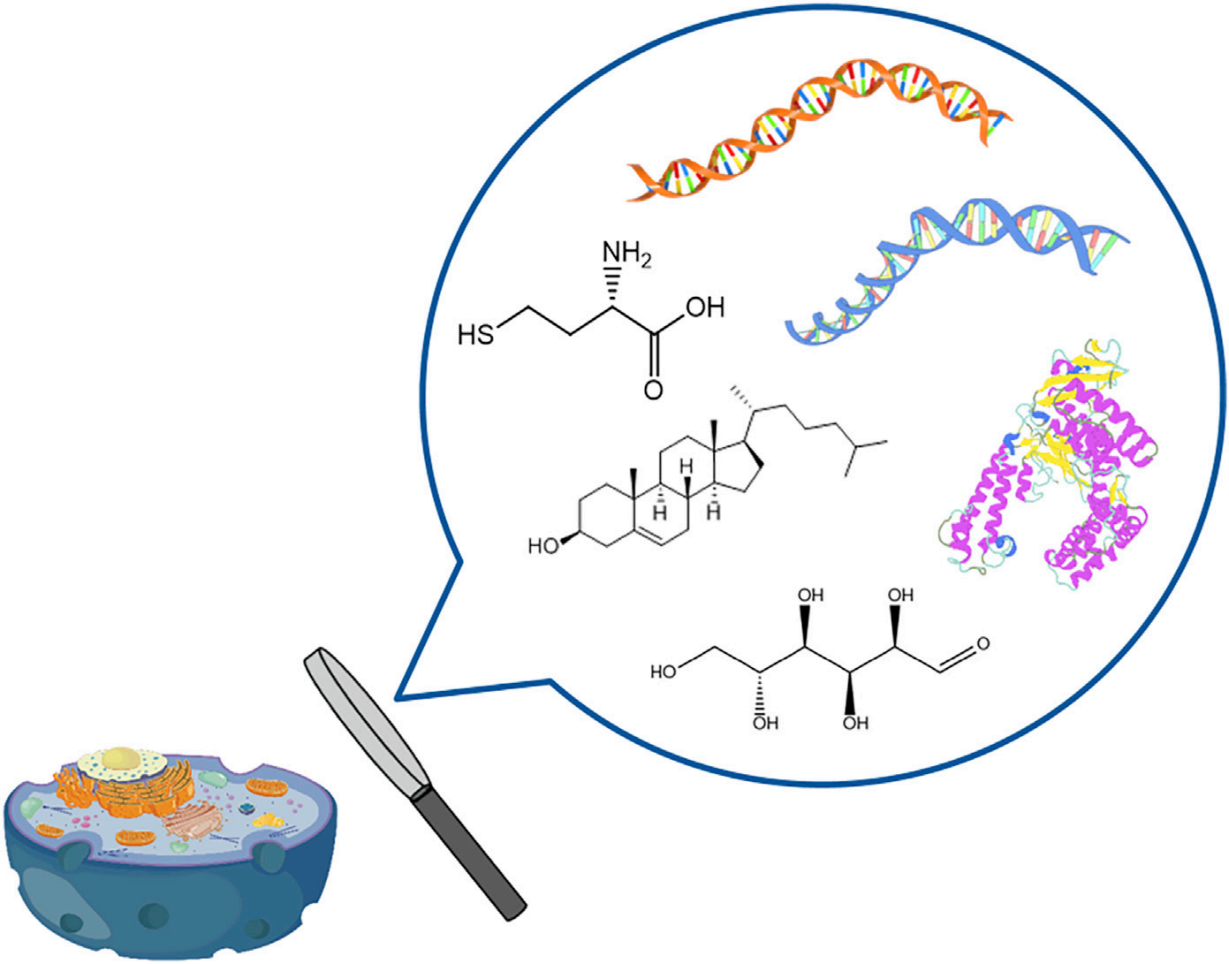
Single-cell multi-omics analysis.

Over the last decade, there have been significant advancements in single-cell analysis technologies (Figure 1). These breakthroughs encompass a wide range of disciplines, including single-cell genomics, single-cell transcriptomics, and single-cell epigenomics analysis. These innovations have played a pivotal role in advancing medical domains like precision medicine and cancer treatment, as well as biological research areas such as tissue and organ development (Zhang and Vertes, 2018a). In single-cell multi-omics research, single-cell genomics and transcriptomics analysis technologies have continued to make new progress due to the amplifiable nature of nucleic acids. Nevertheless, it is important to highlight that single-cell proteomics and metabolomics face considerable hurdles and have not garnered comparable attention or widespread adoption. This is primarily attributed to the formidable challenges arising from the exceedingly low concentrations of target substances and the limitations imposed by conventional analytical methodologies. In particular, single-cell metabolomics lags behind other omics disciplines and eagerly awaits the emergence of novel analytical approaches to bridge this gap.

Recently, mass spectrometry (MS), known for its highly sensitive and specific label-free detection capabilities, has found increasing utility in the realm of unicellular metabolomics and proteomics analysis (Ali et al., 2019; Zhang and Vertes, 2018a; Yin et al., 2019; Tajik et al., 2022). Although there are some other single-cell analysis methods (e.g., electrochemical and fluorescence techniques), they often require the analyte molecules to have specific physicochemical properties (redox activity) or need structural modifications (fluorescent labeling), which greatly limits the application scope of these methods (Li et al., 2016; Armbrecht and Dittrich, 2017; Gu et al., 2020). Compared with other methods, MS can detect hundreds of metabolites without requiring labeling or amplification of the analytes. Moreover, MS can elucidate the structure of unknown compounds by using tandem MS and analyzing the characteristic fragment signal (Kaltashov et al., 2018). Moreover, isotope dilution MS enables precise quantitative analysis of single-cell samples, ensuring the highest metrological quality and direct traceability to SI units (Yin et al., 2018). These excellent properties also enable MS to be well applied in single-cell precise quantitation.

**FIGURE 2:**
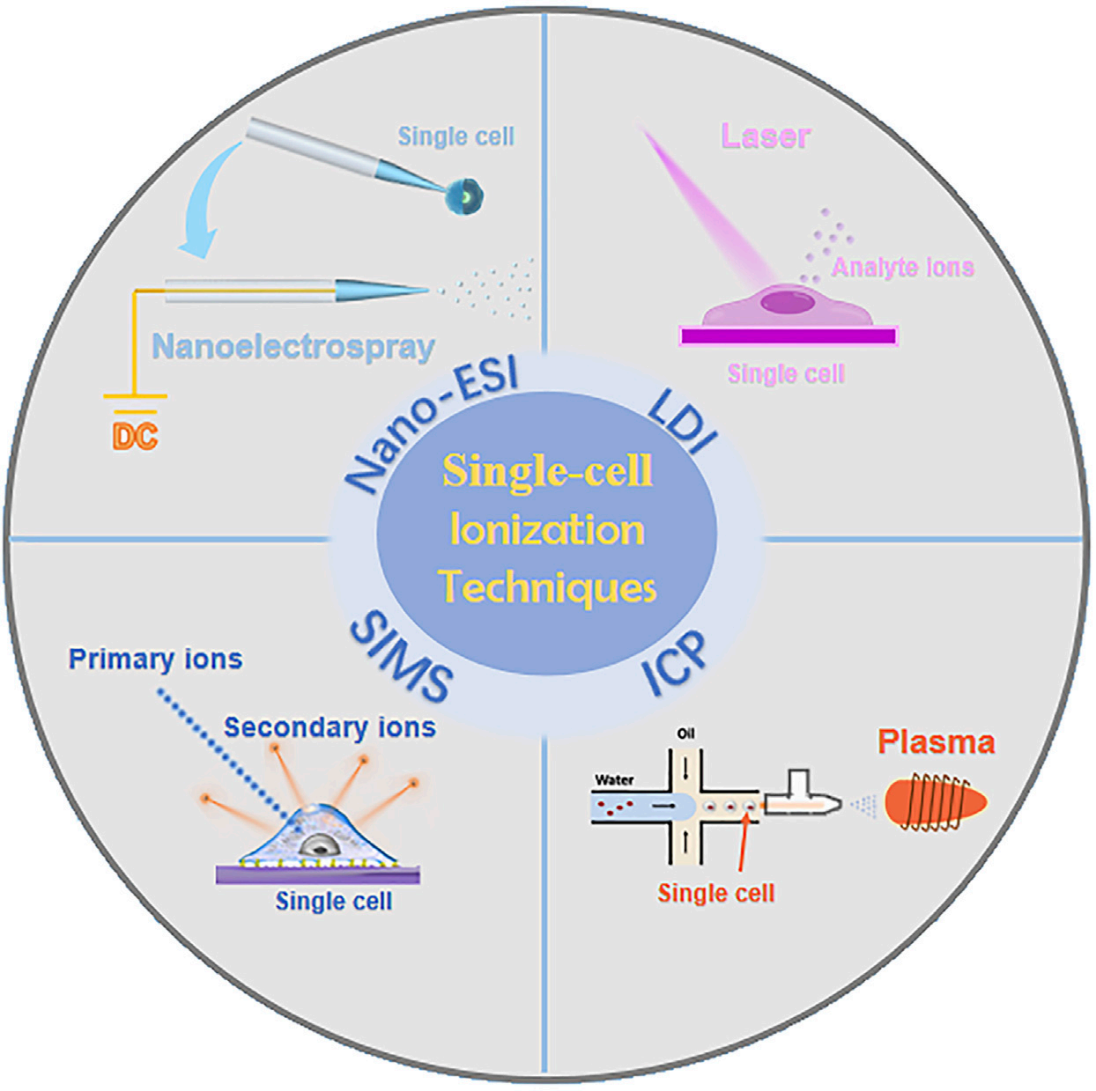
Single-cell MS methodologies based on four ionization techniques.

Currently, researchers have developed single-cell MS methodologies based on diverse ionization techniques (Figure 2), mainly including nano-electrospray ionization (nano-ESI) (Liu et al., 2018; Yin et al., 2018), laser desorption ionization (LDI) (Kompauer et al., 2017), secondary ion mass spectrometry (SIMS) (Hua et al., 2018), and inductively coupled plasma (ICP) (Zhang et al., 2020). The following sections will provide an overview of the respective single-cell MS methodologies based on these four ionization techniques.

## Single-cell MS methodologies based on four ionization techniques

### Single-cell analysis by Nano-ESI

Nano-ESI is a highly efficient and sensitive “soft” ionization technique that is extensively employed in biological analysis and life sciences (Duncan et al., 2019; Lageveen-Kammeijer et al., 2019; Cao et al., 2020). Nano-ESI stands out among other ionization techniques due to its ability for direct, swift, and *in vivo* sampling (Oomen et al., 2019; Yin et al., 2019). Meanwhile, it eliminates the need for specific conditions like matrix and vacuum in the detection process. Nano-ESI typically employs nanospray tip (nano-tip) with inner diameters ranging from 1 to 5 μm for sampling purposes. When augmented with isotope-labeled internal standards and integrated with nano-liquid chromatography (Nano-LC) or capillary electrophoresis (CE), this method excels in achieving precise quantification and characterization of an extensive array of substances, encompassing metabolites, proteins, and lipids within individual cells. Consequently, this approach adeptly addresses the challenges associated with accurate sampling, effective separation of multiple compounds, reliable detection of trace substances, and the mitigation of salt-induced interference at unicellular analysis (Fujii et al., 2015; Root et al., 2017). The extremely low flow rate of nano-ESI (up to 25 nL/min) also provides a longer MS analysis time, which effectively improves the ability of multistage MS to elucidate the structure of unknown compounds (Zhang et al., 2018c). In addition, combining CE and nano-LC can better separate and gradient elute different compounds, eliminate signal interference from impurity molecules, and achieve simultaneous quantification of different compounds in single cells (Cong et al., 2020). In the past few years, driven by advances in analytical techniques and increasing detection demands, researchers have optimized and improved single-cell nano-ESI methods from aspects of sampling, separation, ionization efficiency and structural identification.

#### Sampling

It is of great significance for the development of nano-ESI from the aspect of sampling, arising from its pivotal role in revolutionizing the field of bioanalysis by providing an unprecedented level of precision and accuracy in the measurement of minute single-cell samples. In pursuit of effective sampling at the single-cell level, Zhang’s team (Zhang et al., 2016) introduced a pioneering approach known as single-cell droplet microextraction sampling, leveraging nano-ESI. In this method, the nano-tip initially aspirates the extraction solvent, and then dispenses the solvent droplets onto the cell surface, effectively extracting cellular metabolites into these droplets. Subsequently, the droplets are aspirated back into the nano-tip for further analysis. By altering the solvent and leveraging the varying solubilities of different compounds, this approach achieves a partial separation and detection of diverse compound types, while minimizing interference from salt and culture media matrices. This innovative technique enables the efficient concentration and purification of specific metabolites within individual cells before MS analysis. Feng et al. (Feng et al., 2019) further developed a droplet microextraction array technology on this basis, achieving single cells’ high-throughput quantitative MS detection. The use of microarrays solved the problem of irregular solvent diffusion during direct droplet microextraction, resulting in enhanced cell sorting efficiency and greater control over the sampling process. Through the incorporation of isotope-labeled internal standards, it becomes possible to attain quantitative analysis of carbohydrate metabolites within single cells.

**FIGURE 3:**
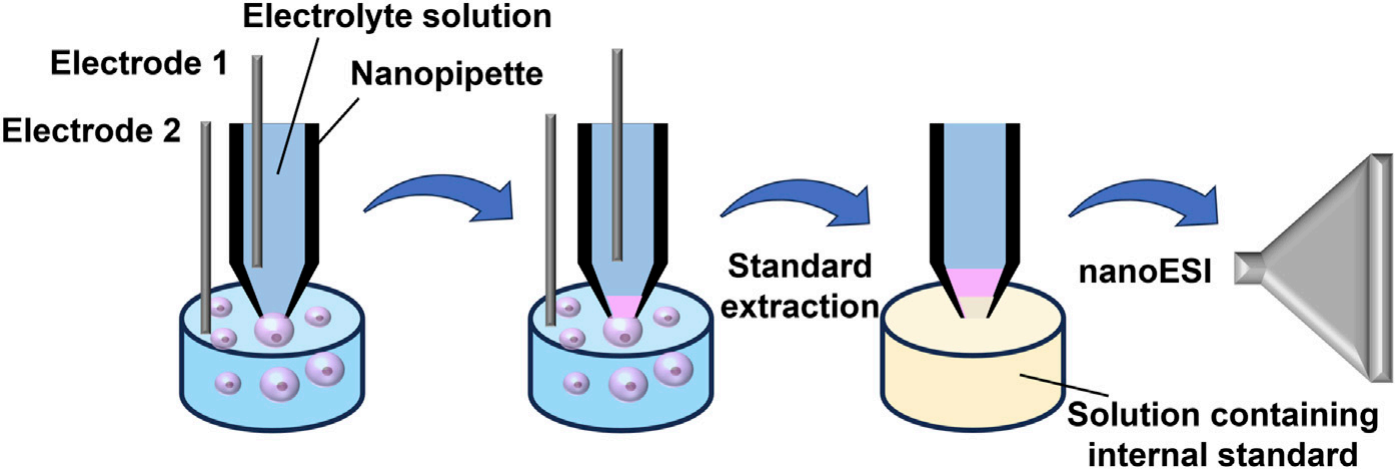
Schematic diagram illustrating Yin et al.‘s method for quantitatively and precisely analyzing molecules in single cells. Adapted with permission from Yin et al. (2018). *Anal. Chem.* 90, 7,937–7,945 (Yin et al., 2018). Copyright 2018 American Chemical Society.

For the precise quantification of single-cell substances, Yin et al. (Yin et al., 2018) employed localized electroosmotic extraction, allowing for the exact control of sub-pL sample volumes extracted from individual cells. This innovative approach utilized a nanopipette with a tip diameter of less than 1 μm and equipped with two electrodes, along with a hydrophobic electrolyte for sample extraction and subsequent nano-ESI detection (Figure 3). The researchers refined the composition of the electrolyte solution within the nano-tip to improve the signal-to-noise ratio (S/N) in MS. By modulating the voltage applied between the two electrodes and adjusting the extraction duration, they achieved precise and quantitative regulation of the extracted sample volume. As a result, they successfully quantitatively extracted 2–5 pL from the cytoplasm of *Allium cepa* cells, enabling the comprehensive detection of over 50 cellular metabolites, including carbohydrates and flavonoids. This platform could not only ensure single-cell physiological activity but also enable the quantitative MS detection of metabolites within individual cells at subcellular levels, which made up for the shortcomings of MS in live sample quantification.

Unicellular metabolites experience instant change upon variations to the surrounding microenvironment, but most nano-ESI methods require a tedious and time-consuming sampling process. Therefore, rapid and online nano-ESI techniques are necessary for live single-cell analysis. Yang’s group (Liu et al., 2018) designed a miniaturized and consolidated sampling and ionization instrument called the T-probe. The T-probe consists of a sampling nano-tip, an MS sampling injection nano-tip, and a solvent supply capillary, integrated in a “T" shape. By manipulating both solvent velocity and electrolytic voltage, a suitable suction force is generated at the sampling probe to withdraw cellular contents. At the T-junction, the solvent mixes with cellular species, and the resulting mixture is then delivered to the nano-ESI emitter for ionization and MS detection. This device facilitates online, *in situ* single-cell MS analysis and metabolic spectrum analysis at the single-cell level under environmental conditions. By enlarging the tip diameter, this group (Zhu et al., 2019) further redesigned an advanced T-probe, which was connected to a mass spectrometer, to facilitate the swift *in situ* analysis of intact, live individual cells in suspension. This innovative design enabled the examination of non-adherent cells while preserving cellular contents, as each cell underwent lysis within the probe. This approach avoided the inherent limitations associated with using cell lysates derived from cell populations. Using this technique, they have recently acquired metabolomics data from individual cells during *T. cruzi* infection, enabling insight into the heterogeneity within infectious diseases (Nguyen et al., 2022).

#### Separation

Efficient separation can ensure the least interference of the target molecule signal during the nano-ESI analysis of single-cell samples. In the pursuit of mitigating mutual interferences among diverse compounds in single-cell analysis, separation techniques like CE and nano-LC have gained widespread adoption. Kawai et al. (Kawai et al., 2019) proposed an improved CE technology to increase the responsiveness of CE-nano-ESI-MS when analyzing particular metabolites within individual cells. This method combined a high-efficiency capillary electrophoresis mass spectrometry ionization device (nanoCESI) with an enrichment system (LDIS) for targeted single-cell metabolomics analysis. NanoCESI has a 10 μm thin wall and a 5–10 μm tapered tip. The slender wall thickness allows sheathless ionization and maximally reduces the flow rate of single-cell analytes, while the tapered end can efficiently ionize single-cell samples through a nanoelectrospray mechanism, with 3.5 times higher sensitivity compared to traditional ESI needles. LDIS is a large-volume dual preconcentration method for single-cell sample enrichment, achieving efficient concentration and purification of diluted samples at low concentrations. Through the coupling of nanoCESI and LDIS, this platform successfully achieved MS quantification of 20 amino acids in HeLa single cells and identified over 40 single-cell metabolites using TOF-MS. The Kelly research group has developed a single-cell proteomic analysis method based on nano-LC (Cong et al., 2020). This method utilizes a self-packed capillary nano-LC column with an inner diameter of 20 μm for the separation of single-cell protein digests. The samples were prepared using the nanodroplet processing in one pot for trace samples (nanoPOTS) platform previously developed by the research group. NanoPOTS is a proteomics sample preparation method that enables the analysis of very small amounts of protein samples, down to single cells. Processes such as cell lysis, alkylation, and enzymatic digestion were all conducted *in situ* within the droplets (Zhu et al., 2018; Williams et al., 2020). With this approach, proteomic analysis of over 300 proteins was achieved in single HeLa cells.

To eliminate the interferences in single-cell analysis, our group has also proposed a polarity-reversed nanoelectrospray ionization (PR-nESI) technique (Gong et al., 2017; Gong et al., 2019), which has been applied to directly and sensitively analyze hydroxylated polycyclic aromatic hydrocarbons (OH-PAHs) in single cells without further purification (Tan et al., 2022). Utilizing this method, cationic metal ions like K^+^ and Na^+^ experience substantial separation due to the applied electric field, resulting in a highly effective reduction of salt-induced interference in the ultimate mass spectrum. Simultaneously, some positively charged high-abundance cell metabolites can undergo significant electrophoretic migration, enhancing the detection sensitivity of low-abundance OH-PAHs that are frequently obscured by background noise. Consequently, our work achieved a 1–2 order of magnitude increase of S/N for OH-PAHs within unicellular samples compared to traditional nano-ESI. The limit of quantifications (LOQs, S/N > 10) towards OH-PAHs is down to amol level.

#### Ionization efficiency

**FIGURE 4:**
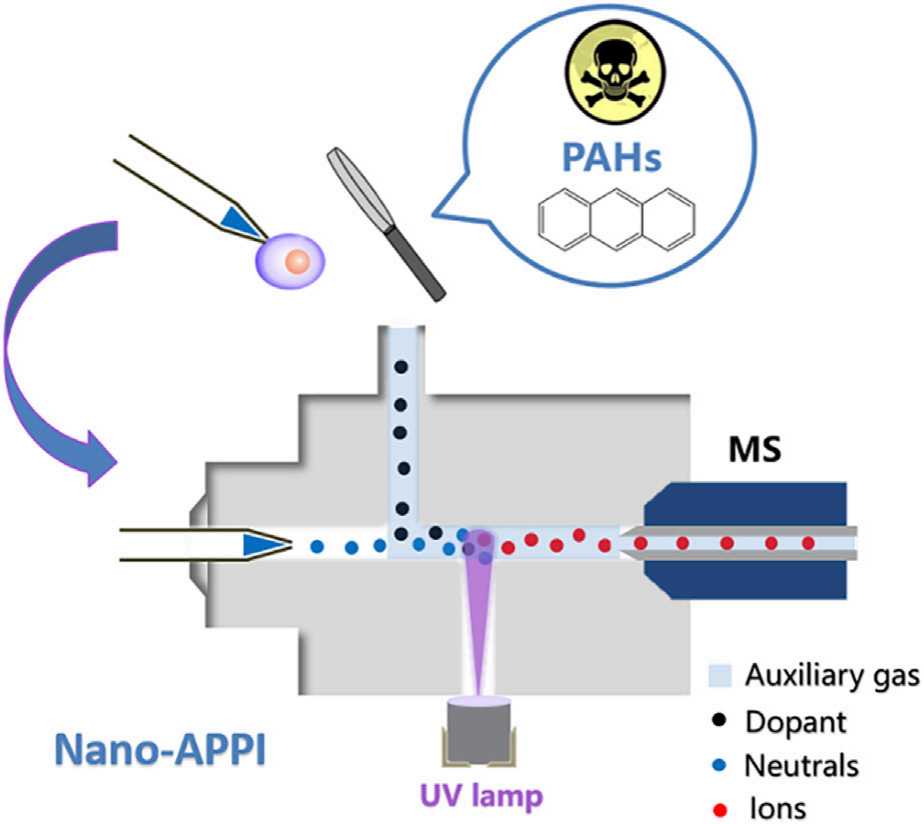
Schematic illustration of nano-APPI for low-polarity PAHs analysis. Adapted with permission from Tan et al. (2023). *Analyst* 148, 3,730–3,739 (Tan et al., 2023). Copyright 2023 Royal Society of Chemistry.

Despite the extensive use of nano-ESI in detecting minute biological compounds within single cells, challenges persist when it comes to nano-ESI’s capability to analyze low-polarity compounds in unicellular samples, primarily due to its low ionization efficiency. Therefore, researchers have developed many new ion source devices by combining nano-ESI with other technologies. These new hybrid ion source devices can realize the high-sensitivity detection of trace compounds (especially low-polarity compounds) in cells or expand the types of detection. Our research team introduced a novel nanoliter atmospheric pressure photoionization (nano-APPI) source (Figure 4) (Tan et al., 2023). This innovation empowers the sensitive detection of PAHs in individual samples. Through the amalgamation of nano-ESI and atmospheric pressure photoionization (APPI) benefits, the nano-APPI source has achieved exceptional responsiveness and can analyze PAHs down to the fmol level. In contrast to the conventional atmospheric pressure chemical ionization (APCI) approach, this method has substantially enhanced the detection threshold for PAHs by 1–2 orders of magnitude. Through the optimization of several parameters, we attained high-efficiency ionization of PAHs, enabling the detection of low-abundance PAHs (down to fmol level) within individual cells. We foresee that this novel approach will offer significant benefits in the examination of PAHs and associated metabolites in unicellular analysis. It will enhance our comprehension of how PAHs impact organisms from a toxicological perspective and provide valuable insights for healthcare practices.

To improve the analytical sensitivity and throughput, Zenobi’s group proposed a high-efficiency, high-throughput, and no-labeling system for single-cell analysis, utilizing a dielectric barrier discharge ionization (DBDI) with an active capillary in which ionization occurs within a capillary linked directly to the MS inlet (Liu et al., 2021). In this work, the researchers initially disintegrated cells using MeOH within the cell introduction system and subsequently ionized the cellular metabolites using a DBDI source. Notably, a DBDI ion source was used in this device, achieving the highly efficient ionization and analysis of various single-cell molecules (especially low-polar compounds). This platform effectively sorted different cells based on their single-cell metabolite profiles in mixed-cell suspensions, categorizing each cell type into specific groups. This capability proved invaluable in probing lipid metabolism in clinical tumor tissues. Since then, Zenobi’s group has continuously improved the method of DBDI and further expanded the application of this method. Building on the foundation of the DBDI source, they developed a hybrid ionization approach that amalgamates nano-ESI with DBDI for the ultrasensitive detection of both polar and low-polarity compounds within individual cells (Liu et al., 2022). Nano-ESI served as the source for ionizing polar metabolites, while DBDI was employed as a post-ionization source to enhance the ionization efficiency of low-polar metabolites in cells that were challenging to ionize with nano-ESI alone. Following optimization, employing ESI at 3.5 kV and DBDI at 2.6 kV, this hybrid ionization method resulted in the detection of 49 additional compounds in onion cells and the identification of 73 more compounds in PANC-1 cells. This integrated ionization source not only broadened the scope, improved ionization efficiency, and lowered the detection limit for metabolites with diverse polarities but also significantly impacted the continually advancing field of single-cell metabolomics.

#### Structural identification

In the realm of nano-ESI analysis focused on single-cell compounds, structural identification is pivotal for understanding the intricate molecular makeup and functional properties of these compounds. Through techniques like tandem MS, researchers delve into elucidating the exact arrangement of atoms, functional groups, and overall molecular architecture within these minuscule samples. This process enables not only the identification of specific compounds but also aids in uncovering their potential biological significance. Yet, nano-ESI commonly captures exceedingly transient MS signals for single-cell samples (pL-level volume), posing challenges for tandem MS analysis and structural identification. To prolong the extremely brief or almost instantaneously detected MS signals from pL-level samples via nano-ESI, Zhang’s group (Wei et al., 2015) introduced a technique known as pulsed direct current electrospray (Pico-ESI), which can significantly extend the MS signal duration, enabling systematical profile and structural identification of unicellular metabolic components in minute sample volume. Zhang’s research team (Zhang et al., 2018c) extended their work by incorporating Pico-ESI-MS with the droplet microextraction technique, enabling efficient high-throughput metabolomics analysis in the scale of individual cells. This integrated approach generated a comprehensive dataset comprising more than 600 tandem mass spectra (MS^2^) of metabolites extracted from individual mammalian cells, successfully identifying over 300 phospholipids in the process.

**FIGURE 5:**
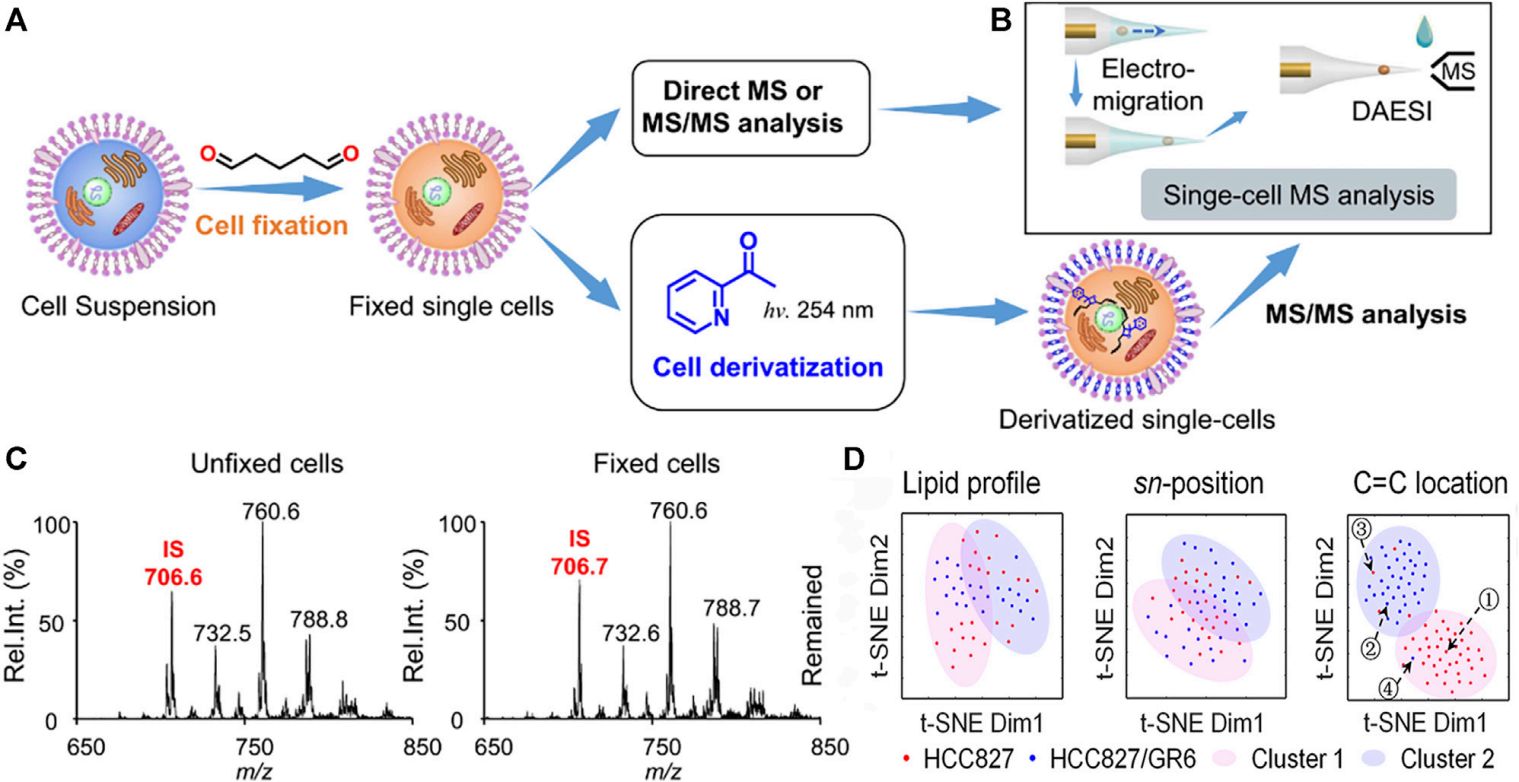
The workflow of highly structurally specific lipidomic analysis in single-cell analysis. **(A)** Procedure for fixing individual cells and optional derivatization for subsequent MS analysis. **(B)** Method for single-cell MS analysis utilizing electromigration and DAESI, enabling multiple rounds of sampling and data acquisition. **(C)** Precursor ion scan of *m/z* 184 spectra for extracts from unfixed (left) and fixed (right) MDA-MB-231 cells. Internal standard (IS): PC 15:0–15:0 (*m/z 706*). **(D)** The distinction between gefitinib-sensitive and gefitinib-resistant human lung cancer cells: *t*-SNE plot comparing molecular lipid profiles, *sn*-position isomer compositions, or C=C location isomer compositions of HCC827 and HCC827/GR6 single cells. Adapted with permission from Li et al. (2021a). *Nat. Commun.* 12, 2,869 (Li et al., 2021a). Copyright 2021 Springer Nature.

In recent years, single-cell lipidomics has emerged as a vital field, supplementing other omics approaches by providing a unique lipid-centric perspective within individual cells (Zhang et al., 2022; Wang et al., 2023). Xia’s group (Ma and Xia, 2014) proposed a method combining the Paternò-Büchi (PB) reaction and tandem MS to identify the C=C position in lipid fine structures. The results revealed that UV irradiation of a nanoelectrospray plume, which entrained lipids and acetone, enhanced the PB reaction. Tandem MS of the online reaction products, through collision activation, led to oxetane ring rupture and the formation of diagnostic ions specific to the double bond’s location. This method enabled confident, rapid, and sensitive determination of double bond positions in various lipids (Ma et al., 2021). Based on this, Zhu and others (Zhu et al., 2020) devised a novel technique utilizing a micropipette needle. This method induces PB reactions at C=C bonds, enabling the determination of C=C bond positions in unsaturated lipids at the single-cell level. This innovative approach holds promise for enhancing molecular analysis across diverse single-cell studies, broadening the scope of reactive single-cell MS investigations. Ouyang’s research group (Li et al., 2021a) developed a general approach to enable highly structurally specific lipidomic analysis on the individual cell scale (Figure 5). To realize the PB chemical derivation of single cells in batches, they used glutaraldehyde to fix cells and retain lipids. The combination of electromigration and droplet-assisted electrospray ionization (DAESI) streamlined the MS process in unicellular analysis, rendering it not only straightforward but also exceptionally efficient in terms of sample consumption (Li et al., 2020). The electromigration process enabled cells to focus on the front end of the tip, while the DAESI step could effectively extract and ionize lipids from the single cell. Because the cells were focused at the front end, only a very small amount of liquid droplets were needed to achieve the extraction and minimize the dilution of the substance. Subsequent quantitative analysis of lipid double bond location and stereochemistry isomers in approximately 160 cells allowed the correct classification of four unique subtypes among human breast cancer cells. Crucially, this in-depth single-cell lipidomics approach effectively discriminated against cells resistant to gefitinib within a population of wild-type human lung cancer cells. This underscored the exceptional potential to advance the field of precision medicine.

### Single-cell analysis by LDI

LDI represents a “gentle” ionization method that employs a laser with a designated energy and wavelength for irradiating the target sample, leading to desorption and ionization. For this process to occur, the sample molecules must absorb the laser’s energy at this specific wavelength (Trouillon et al., 2013; Yin et al., 2019). Matrix-assisted laser desorption/ionization (MALDI), in particular, employs an additional matrix material mixed with the analytes, achieving wide molecular detection with little ion fragmentation. LDI and MALDI both use lasers to excite solid samples to generate gaseous ions, but their application ranges are different (Peterson, 2006). LDI is often used to analyze inorganic salts, elements, dyes, or molecules with high light absorption characteristics, and it is not suitable for analyzing biological macromolecules with extremely low volatility. MALDI is suitable for the analysis of non-volatile solid or liquid analytes, especially for ionic or polar analytes. Compared with nano-ESI, LDI-MS has greater capabilities for on-site unicellular analysis, eliminating the requirement for robotic sampling and enabling exceptional throughput unicellular genomics imaging analysis (Ya and Liu, 2017; Zeng, 2018). In recent years, researchers have effectively developed novel LDI-MS methods in unicellular analysis, achieving exceptional throughput, high spatial resolution, low fragmentation, and high ionization efficiency by building new laser platforms, developing single-cell sampling devices, and preparing efficient matrix molecules.

**FIGURE 6:**
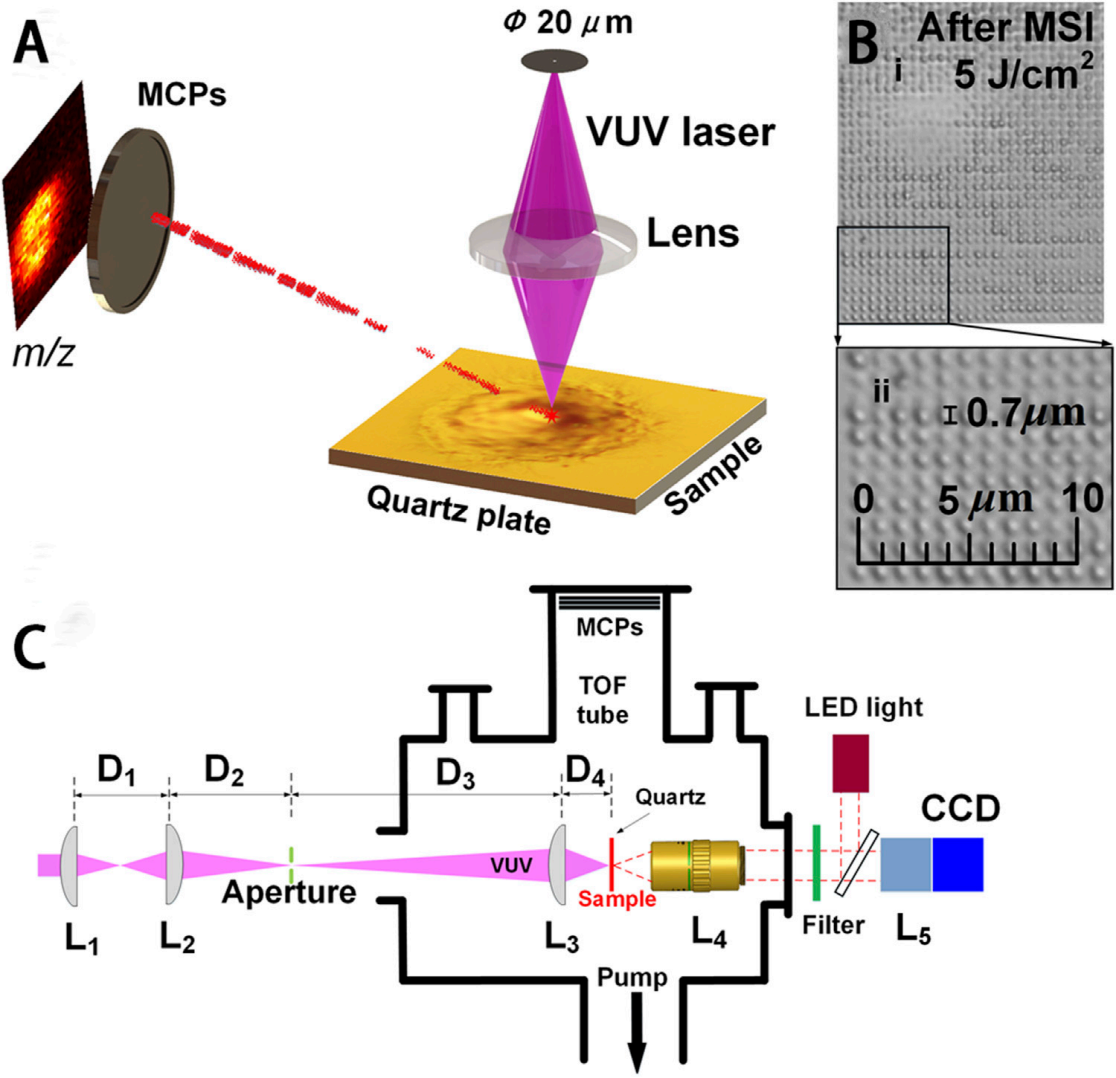
VUVDI-MS platform for unicellular imaging. **(A)** VUVDI-MS concept for submicron laser spot. **(B)** i, HeLa cell imaged via VUVDI-MS; ii, magnification of the black box. **(C)** Schematic illustration of the experimental setup for focusing and characterizing the VUV laser beam. Adapted with permission from Wang et al. (2018a). *Anal. Chem.* 90, 10,009–10015 (Wang et al., 2018a). Copyright 2018 American Chemistry Society.

A new MS imaging technique of vacuum ultraviolet laser desorption/ionization (VUVDI) was reported by Wang et al. This technique offers superior spatial resolution and reduced molecular fragmentation when compared to MALDI-TOF MS and TOF-SIMS at the submicron level (Figure 6) (Wang et al., 2018a). By narrowing the VUV laser beam’s spread angle and increasing the precision of optics, VUVDI obtained the reduced patch size of the ablation craters, reaching dimensions below 100 nm. In addition, the specific position of another VUV laser significantly enhanced sensitivity and widened the mass range. The platform was further applied for the imaging of HeLa cells exhibiting craters with diameters measuring 510 ± 80 nm. The results illustrated the successful detection of various cholesterols and phospholipids signals in individual HeLa cells by VUVDI, which were more sensitive than the TOF-SIMS method. Similarly, Wang and others (Wang et al., 2018b) also reported a VUVDI method, utilizing it to visualize intact cholesterol within a tissue section of a mouse esophagus and in mouse zygotes. For the mouse zygotes, this method was utilized to generate MS images depicting the [M – H + H_2_O]^+^ cholesterol fragment under different scanning configurations. And ion fragments originating from amino acids (*m/z* 60), lipids (*m/z* 84), and cholesterol (*m/z* 352) were also conducted by VUVDI-MS, which illustrated the predominant distribution of cholesterol at the zygote boundaries.

**FIGURE 7:**
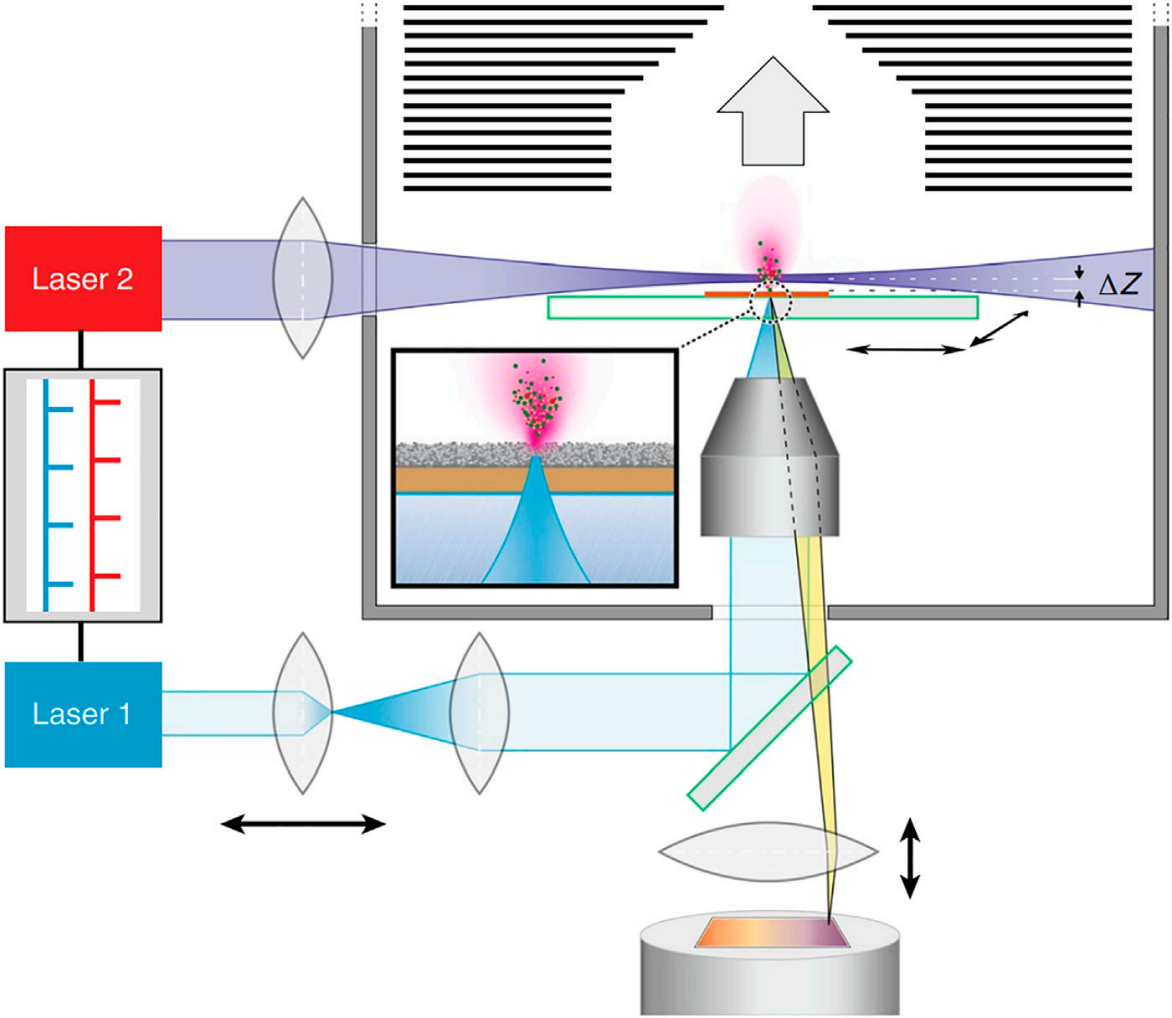
Schematic illustration of t-MALDI-2-MSI. Adapted with permission from Niehaus et al. (2019). Nat. Methods 16, 925–931 (Niehaus et al., 2019). Copyright 2019 Springer Nature.

Despite numerous advancements and applications, the challenge of achieving high-resolution and information-rich MS imaging in unicellular analysis under conditions resembling physiological environments persists. Kompauer and others introduced an atmospheric pressure matrix-assisted laser desorption/ionization mass spectrometry imaging (AP-MALDI-MSI) technique. Attaining a remarkable lateral resolution of up to 1.4 μm, a superior mass resolution exceeding 100,000, and an exceptional precision within 2 ppm was accomplished by this method (Kompauer et al., 2017). The platform seamlessly integrated an advanced focusing objective featuring a numerical aperture of 0.9 at 337 nm, along with a generous working distance of 18 mm. This was achieved within a coaxial arrangement with an Orbitrap mass spectrometer, all while refining the procedure for applying the matrix. The result demonstrated the enhancement of image comparison, lateral resolution, and ion yields compared with up-to-date MSI sources. The method enabled the label-free examination of lipid, metabolite, and peptide distributions at the subcellular level, permitting the differentiation of cilia from the oral groove in *Paramecium caudatum*. Niehaus and others (Niehaus et al., 2019) have developed a matrix-assisted laser desorption-ionization mass spectrometry imaging in transmission-mode geometry (t-MALDI-MSI). This cutting-edge approach enables the visualization of individual cells and tissues with subcellular precision, providing molecular data with a pixel size as small as 1 μm. To address the challenge of reduced ion abundance at smaller pixel dimensions, they adopted a laser-induced postionization (MALDI-2) method. This innovation involved the integration of a t-MALDI-2 ion source with an Orbitrap mass analyzer (Figure 7). Compared with Kompauer’s AP-MALDI-MSI, the laser post-ionization approach greatly improved the crucial sensitivity and accuracy for analysis by a substantial margin, especially for various categories of phospholipids and glycolipids within cultured Vero B4 cells. Recently, Soltwisch et al. utilized t-MALDI-MSI to gain deeper insights into intracellular lipid distribution and uncover molecular characteristics within subcellular regions (Bien et al., 2022). Utilizing statistical tools and machine learning, t-MALDI-2-MSI data was harnessed for the identification of subcategories within the cell culture.

The efficient and highly sensitive profiling of individual cells plays a pivotal role in advancing our understanding of life systems at the unicellular and subcellular levels. To enhance the throughput of MALDI-MS, Urban and others (Urban et al., 2010) proposed a highly concentrated micro-arrays for mass spectrometry (MAMS) technique, enabling swift pL-volume aliquoting and ultra-sensitive analysis for yeast cells (*Saccharomyces cerevisiae*). Based on the high sensitivity of MALDI-MS, MAMS made up for the low-throughput characteristic of the traditional MALDI method and realized high-throughput detection of a wide array of molecules within individual cells. MAMS was further applied for the detection of metabolites and compounds of biological interest (bioactive peptides, a drug, and an intact protein) on the unicellular scale. Although MAMS achieved exceptional throughput unicellular analysis, the variation in the cellular environment and the invasive extraction of cellular contents could cause interference and damage to the analytes in cells, thereby changing the initial state of unicellular samples and reducing the detection accuracy for MALDI-MS. Therefore, Guillaume-Gentil and others (Guillaume-Gentil et al., 2017) proposed a technology combining fluidic force microscopy (Fluid FM) and MAMS. This approach allows for the lossless and quantitative sampling of cellular contents for the subsequent MALDI-MS analysis. The FluidFM probe had a smaller size and a favorable pyramid structure avoiding membrane destruction. Additionally, the probe was actuated using an atomic force microscope (AFM), which could accurately and gently quantify the intracellular analytes. This method could minimize physiological interference and maintain the original cellular environment, allowing the characterization of 20 metabolites in an individual HeLa cell.

**FIGURE 8:**
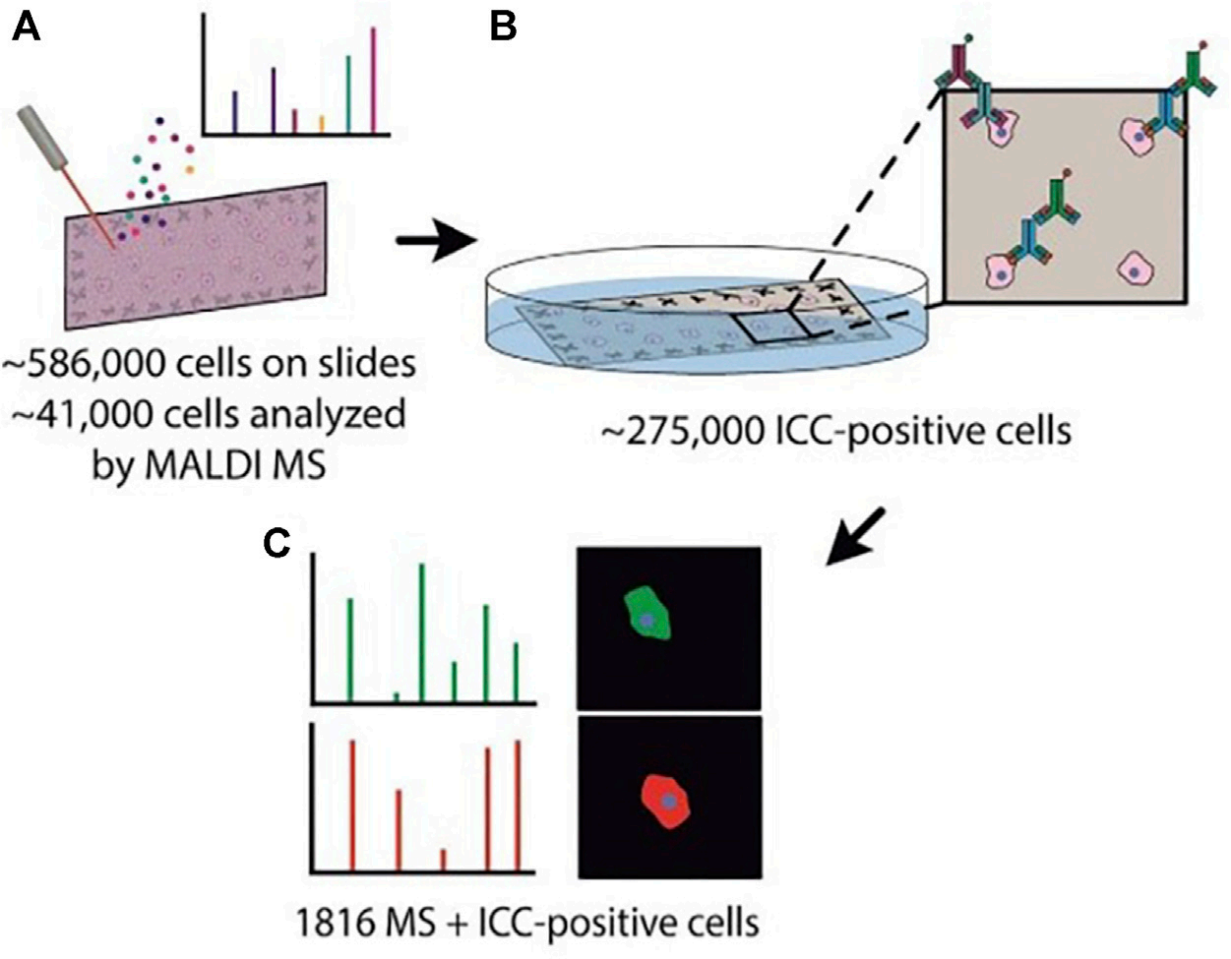
Schematic illustration of the MALDI-MS/ICC strategy. **(A)** The localization of brain cells based on microMS and single-cell MALDI-MS analysis. **(B)** Immunostaining process based on ICC. **(C)** Mass spectra and ICC profiles. Adapted with permission from Neumann et al. (2019). *Angew. Chem. Int. Ed.* 58, 5,910–5,914 (Neumann et al., 2019). Copyright 2019 Wiley Online Library.

MALDI-MS is a competitive means for the comprehensive and sensitive measurement of chemical contents in unicells, but MS spectra are challenging for the correlation of cellular type due to the limited metabolic information. Neumann et al. (Neumann et al., 2019) introduced a novel approach that merges MALDI-MS with immunocytochemistry (ICC) to analyze more than a thousand individual rat brain cells. The method allowed exceptional throughput MALDI-MS analysis of thousands of single cells which were subsequently differentiated by ICC (Figure 8). MicroMS, a cell-finding and analysis software, was employed for the localization of ∼586,000 cells. Then unicellular MALDI-MS was conducted on approximately 41,000 structures resembling cells, each of which was at least 100 μm away from neighboring cells. Following the MS analysis, the MALDI matrix was eliminated, and the cells were preserved using a 4% PFA solution in a phosphate-buffered solution. Afterward, immunostaining targeting GFAP and NF-L was conducted. Although GFAP is not a universal astrocytic marker, the majority of astrocytes in the cerebellum can be positively identified through GFAP immunolabeling. Similarly, NF-L antigens are expressed in most neurons. Cells that tested negative in ICC might either belong to different cell types lacking these antigens or represent damaged cells. Because of their unclear identification, ICC-negative cells were excluded from the mass spectral analysis. So immunostaining was successfully performed on approximately 275,000 cells on the slide and further applied a comprehensive MS and ICC profile in single cells. The performance of ICC after MALDI-MS widely expanded the scope of chemical identification, such as lipids and small metabolites, contributing to a deeper understanding of brain functionality. Although most lipid contents exhibited a high similarity between neurons and astrocytes, there were notable differences observed between these 2 cell types. This method could provide abundant chemical information and precise cell classification through MALDI-MS/ICC on thousands of cells, allowing the deep exploration of the diversity among cells in the mammalian brain.

### Single-cell analysis by SIMS

SIMS, renowned for its exceptional sensitivity and impressive resolution, stands out as a widely employed ionization technique for surface analysis (Svatos, 2011; Lanni et al., 2012). SIMS normally uses a specific ion beam (primary ions) directed at the sample’s surface, leading to the emission of intact molecules, molecular fragments, and atoms. This process results in the emission of intact molecules, molecular fragments, and atoms, subsequently transformed into charged ions (secondary ions). The charged ions are separated and detected by a mass spectrometer to obtain chemical information at a depth of 1–2 nm on the surface. This method has the functions and advantages of high surface sensitivity, isotope detection, trace component detection, and spatial molecular distribution information detection. It is applied to the imaging analysis of metals, organic compounds, salts, etc. Compared with LDI, SIMS has a smaller desorption site, making it capable of generating high-resolution distribution maps of elements or small molecules at a sub-50 nm scale. Furthermore, SIMS has found extensive use in three-dimensional mass spectrometry imaging (MSI) research, showing significant promise in life sciences, particularly for single-cell imaging analysis (Athwal and Lombaert, 2019). However, compared with nano-ESI and LDI, SIMS is a “harder” ion source, and its ionization intensity is stronger than nano-ESI and LDI. As a result, its high-energy primary ion beam easily leads to excessive fragmentation of sample molecules and complex mass spectra, which makes it difficult to analyze biomacromolecules in single cells completely. In recent years, with the increasing demand for unicellular SIMS analysis, researchers have made improvements in the primary ion beam, optimized image processing techniques, and implemented delayed extraction of secondary ions (Vanbellingen et al., 2015; Passarelli et al., 2017; Vollnhals et al., 2017). These developments have enabled efficient ionization and high-definition imaging analysis of macromolecules within individual cells.

**FIGURE 9:**
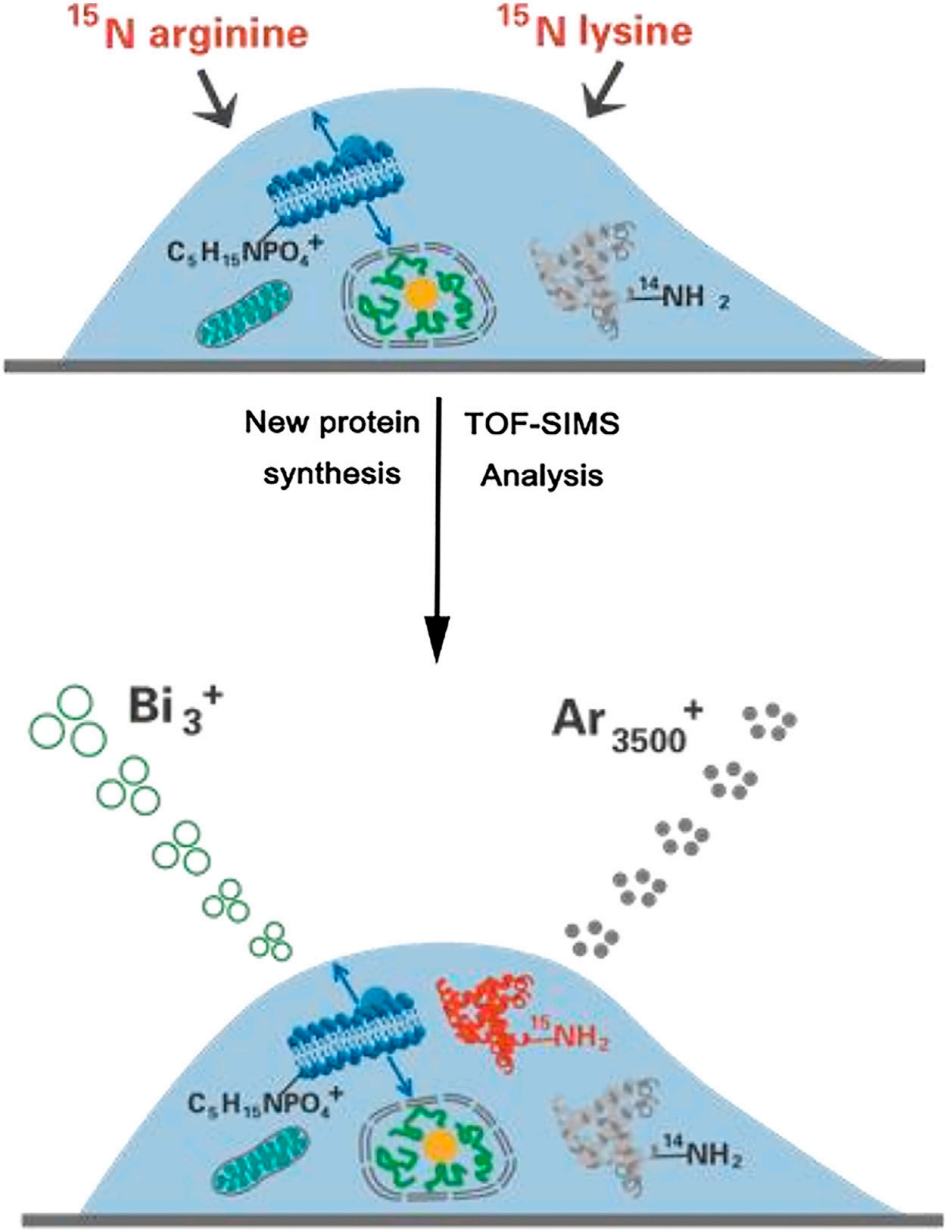
Schematic illustration of simultaneously visualizing proteins and lipids using TOF-SIMS. Adapted with permission from Sheng et al. (2017). *Int. J. Mass Spectrom.* 421, 238–244 (Sheng et al., 2017). Copyright 2017, Elsevier B.V.

The MSI capability of SIMS makes it widely used for tracking small molecules on the unicellular scale. However, the traditional SIMS utilized a high-energy atomic ion beam for sample bombardment, resulting in severe ion fragmentation and low spatial resolution. Tian et al. (Tian et al., 2017) developed a 3D imaging cluster TOF-SIMS approach for examining the chemical distribution of small molecules within both aggregated and individual *E. coli* cells. This label-free method employed a C_60_^+^ primary cluster ion gun to localize endogenous and exogenous small molecules in individual cells, enhancing ion yield while minimizing cumulative damage. This groundbreaking method was effectively employed to map non-labeled intact antibiotics, specifically ampicillin (AMP) and tetracycline (TET), within unicellular *Escherichia coli*. Furthermore, it delved into the impact of antibiotics on these single cells. The results yielded compelling evidence showcasing a notable reduction in tetracycline accumulation within an *E. coli* strain that featured the tetracycline-specific efflux pump (TetA) when contrasted with the isogenic control. For tracking macromolecules on the unicellular scale, Sheng et al. (Sheng et al., 2017) presented a method that enabled the concurrent imaging of recently generated proteins and distinct lipids during the growth of individual HeLa cells. This was accomplished using TOF-SIMS in combination with the metabolic integration of amino acids labeled with ^15^N (Figure 9). By treating single HeLa cells with ^15^N-labeled arginine and lysine, newly formed proteins can be marked with ^15^N. TOF-SIMS is capable of achieving submicrometer resolution mapping of these newly formed proteins and specific lipids by detecting fragment ions of ^15^N-labeled proteins and fragment ions of sphingomyelin (C_5_H_15_NPO_4_^+^) or cholesterol (C_27_H_45_^+^). For image acquisition, the cell was scanned using a Bi_3_^+^ liquid metal ion gun, while Ar_3500_^+^ was employed to perform sputtering on the cell for depth profiling. The statistical results from repeated experiments illustrated that specific lipids exhibited extensive distribution in the cleavage furrow during cellular division, while only a limited number of newly synthesized proteins were observable in this area. This TOF-SIMS technique achieved a deep understanding of the behavior of intact proteins and their spatial distribution within individual cells.

**FIGURE 10:**
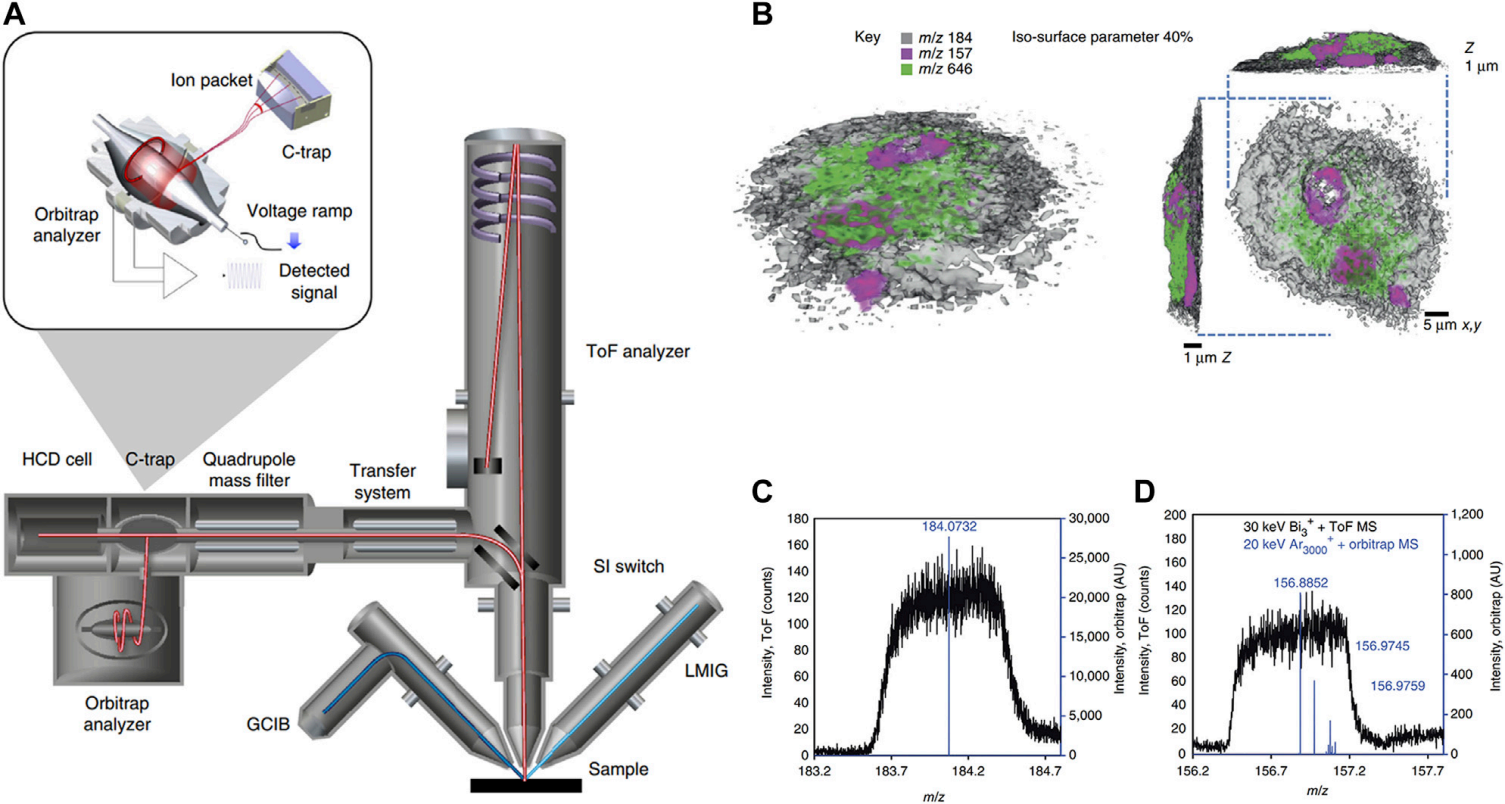
Schematic diagram and imaging results of 3D OrbiSIMS spectrometer. **(A)** The schematic illustration of 3D OrbiSIMS spectrometer. **(B)** 3D MS imaging of individual rat macrophage cell treated with the drug, using dual beam and dual spectrometer. Rendering of the cell with phosphocholine marker, (*m*/*z* 184, gray, opacity 0.40), nuclear marker (*m*/*z* 157, magenta) and amiodarone [M + H]^+^ (*m*/*z* 646), green. **(C)** The high-mass-resolution spectrum from sputtered material using Orbitrap MS (blue) and low-mass-resolution spectra obtained during imaging with the TOF MS (black) for phosphocholine marker. **(D)** As in C for nuclear marker. Adapted with permission from (Passarelli, et al., 2017). Springer Nature, copyright 2017.

Although TOF-SIMS can capture 3D images, it often lacks the essential mass resolution and precision to acquire chemical components and structures. Passarelli and others (Passarelli et al., 2017) introduced a 3D OrbiSIMS device, which provided label-free imaging of metabolites with exceptional mass-resolving capability and subcellular lateral resolution (Figure 10A). This instrument ingeniously combines the remarkable spatial resolution of SIMS with the exceptional mass-resolving capability of an Orbitrap mass spectrometer. To achieve subcellular-level imaging of individual cells, the equipment was employed in a dual beam and dual spectrometer mode. This innovative approach utilized the Bi_3_^+^ liquid metal ion gun in tandem with TOF acquisition to capture high-spatial-resolution images. Furthermore, it incorporated an argon gas cluster ion beam sputtering cycle between images to obtain high-resolution mass spectra using the Orbitrap analyzer. With a high degree of spectroscopic confidence, this method successfully achieved spatial mapping of neurotransmitters within the mouse hippocampus at subcellular scales. Twenty-nine sulfoglycosphingolipids and fifty-five glycerophospholipids could also be putatively identified and mapped by tandem MS. The result also showcased single-cell metabolomics profiling conducted on rat alveolar macrophage cells exposed to varying doses of the drug amiodarone. This led to the identification of an increase in phospholipid species and cholesterol levels, which was ascribed to the accumulation of amiodarone (Figure 10B). The high mass-resolved power facilitated the precise identification of various species, including the phosphocholine marker, nuclear marker, and amiodarone (Figures 10C,D).

A specific variation of SIMS, known as gas cluster ion beam secondary ion mass spectrometry (GCIB-SIMS), has showcased remarkable capabilities in mapping the predominant intact lipids commonly found in cellular biomembranes. By harnessing GCIB-SIMS imaging with a 70 keV (H_2_O)_n_^+^ (n > 28,000) cluster ion beam, Bayır et al. (Sparvero et al., 2021) achieved the mapping of peroxidized phosphatidylethanolamine species. These species are significant predictive biomarkers for ferroptosis and were imaged with an exceptional spatial resolution of 1.2 μm. This imaging was conducted within ferroptotic H9c2 cardiomyocytes and cortical/hippocampal neurons following traumatic brain injury. Importantly, these observations were made at the level of individual cells or within subcellular regions. This result demonstrated that implementing this protocol enabled the visualization of peroxidized lipids at physiologically relevant levels (20 pmol/mmol lipid) in subcellular compartments and their accrual under pathological circumstances.

**FIGURE 11:**
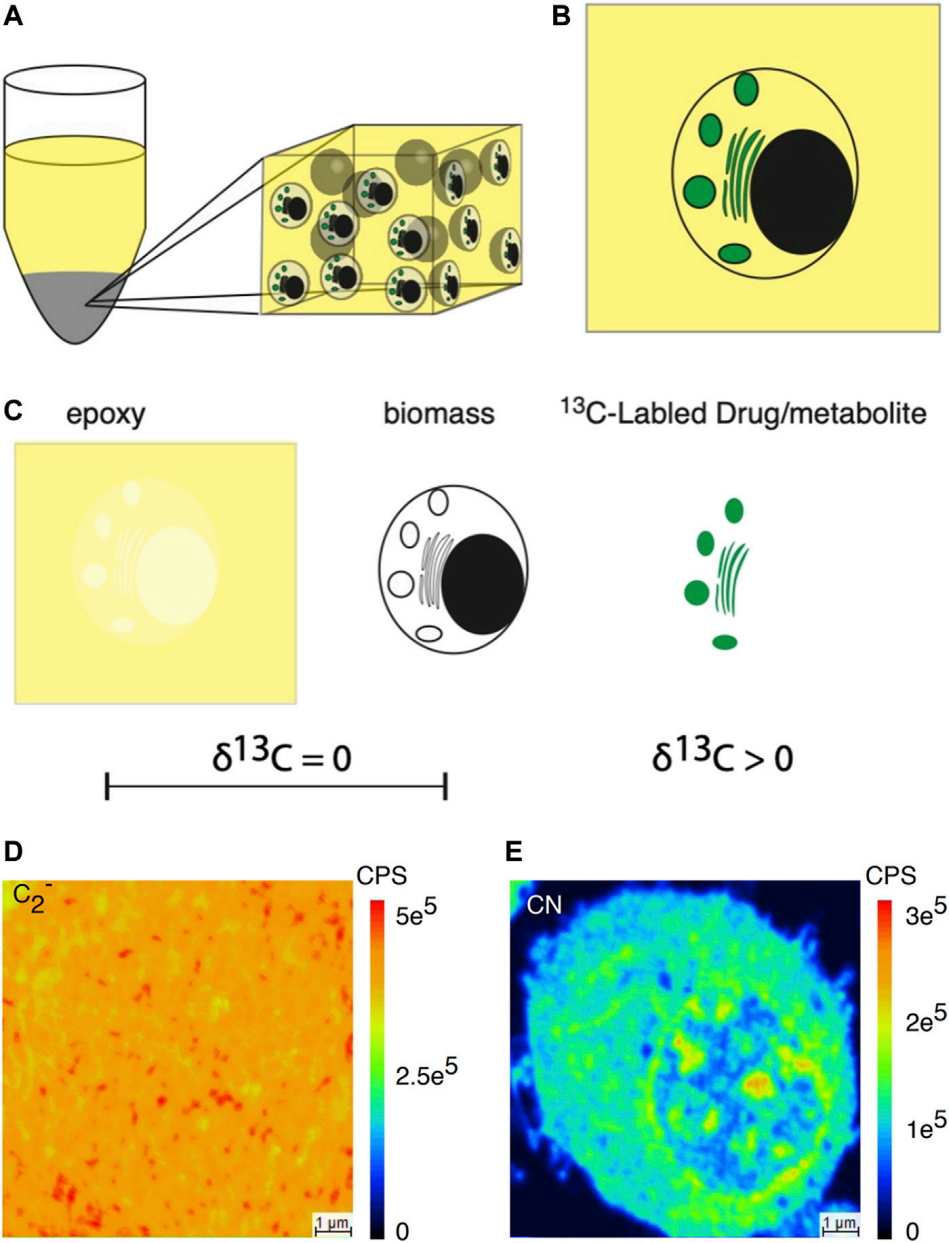
Schematic illustration of subcellular nanoSIMS imaging and absolute quantitative analysis of drugs and metabolites within individual cells. **(A)** Diagram illustrating a resin-embedded cell pellet. **(B)** Diagram representing a section from a resin-embedded cell pellet. **(C)** The three primary carbon sources in the resin-embedded cell material: are epoxy, biomass, and ^13^C-labeled drugs or metabolites. Epoxy and biomass lack ^13^C enrichment, while the labeled drug introduces ^13^C enrichment. **(D)** Secondary ion image of ^12^C_2_^−^. **(E)** Secondary ion image on CN^−^ across the resin-embedded cell material. The δ^1^³C value refers to the deviation of the isotopic ratio in parts per thousand (‰) relative to a reference, typically the Vienna Pee Dee Belemnite (VPDB). Adapted with permission from Thomen et al. (2020). ACS Nano 14, 4,316–4,325 (Thomen et al., 2020). Copyright 2020, American Chemical Society.

By introducing the exogenous isotopic labels, it is now feasible to contrast and gauge the chemical compositions of single organelles about unprocessed or standardized ion signals for single-cell MS analysis. Nevertheless, the relative quantification method is largely dependent on perturbing intact cells and tissues, which poses significant challenges in evaluating the effects of these disturbances on the measurements. Thomen et al. (Thomen et al., 2020) proposed a nanoSIMS imaging approach that achieved precise quantification, as opposed to relative quantization, of carbon species in the subcellular imaging of drugs and metabolites (Figure 11). In this study, the researchers combined epoxy resin, biomass, and ^13^C-labeled drugs or metabolites to create carbon materials. They underscored that the isotope enrichment was exclusively attributed to the presence of ^13^C-labeled drugs or metabolites amid these components. The foundation of this work was that the epoxy resin was utilized to encapsulate the analyzed cells, which closely matched the carbon content within cell biomass. This allowed the use of the complete flush bonding biomass as a substrate for analysis. This was proved by the nanoSIMS image, which showed minimal change in carbon ion emissions between the epoxy and the flush bonding biomass. The result showed that the measured concentration in resin-coated cellular material accurately represented the content present in cell substructures before sample preparation. The present quantitative nanoSIMS method was further applied to PC12 cells, revealing that the dopamine concentration in individual cell vesicles fell within the range of 60 mM, consistent with the results obtained from electrochemical measurements.

Spatial metabolomics imaging has the potential to reveal intercellular variability and tissue arrangement. Zhang et al. (Yuan et al., 2021) developed a spatial single nuclear metabolomics (SEAM) methodology, which is known as the space mononuclear metabolomics technique to dissect organizational microenvironments at the individual cell level. SEAM has two main components: SIMS imaging analysis and a computational analysis suite. This suite enables the visualization of multiscale and multicolor tissue tomography, as well as the recognition and grouping of individual nuclei according to their *in situ* metabolic profiles. In this work, they investigated the contour of spatial metabolism within the liver affected by fibrosis in humans. They detected distinct metabolic traits among groups of hepatocytes, influenced by their proximity to the fibrotic region. This revelation was subsequently validated using spatial transcriptomics via Geo-seq. The capacity of SEAM in unicellular analysis will enhance our comprehension of tissue metabolic organization.

### Single-cell analysis by ICP

ICP is an advanced ionization technique that employs high-temperature plasma to ionize samples. When coupled with MS, ICP-MS allows simultaneously determine and quantify various metals (Fe, Mn, Zn, Cu, Co, etc.) and several nonmetals (P, S, B, etc.) (Liu et al., 2014; Yu et al., 2020). It excels in the precise analysis of elements, especially rare metal elements, compared with the above three methods. ICP-MS offers various advantages as an elemental detector, including its ability to achieve low detection limits, minimal matrix effects, the capacity for multi-element detection, extensive kinetic scopes, and exceptional spectral resolution in both elements and isotopes (Bings et al., 2010; Pröfrock and Prange, 2012). Thanks to these unique properties, analyzing individual cells using ICP-MS is commendable, enabling the investigation of metalloproteins, metalloenzymes, nucleic acids, and some other molecules with an elemental tag (Liu et al., 2017). Till now, ICP-MS has found widespread application in the study of nanoparticle uptake, labeling, and tagging policies, mapping the distribution of elements in tissues, and the generation of quantitative data for biomedical research. It has also been effectively employed alongside molecular MS techniques in biological systems, serving as a complementary analytical tool (Pozebon et al., 2017).

With high throughput and low limit of detection, flow cytometry has been widely applied for decades in biological fields, enabling the systematic analysis of multiple features in individual cells (Davey and Kell, 1996; Krutzik et al., 2008). Mass cytometry (MC) represents a fusion of ICP-MS precision with the remarkable throughput capabilities of flow cytometry, empowering the investigation of intricate cellular systems and the detection of low-abundance species at the unicellular level (Bandura et al., 2009; Bendall et al., 2011; Orecchioni et al., 2017; Ajami et al., 2018; Levine et al., 2021). Unlike fluorophore emission spectra overlap in flow cytometry, the ICP-MS can clearly distinguish and quantify rare-earth element isotope signals, allowing the precise single-cell analysis of various analytes with rare-earth element tags (Van Acker et al., 2023). MC possesses unique superiority in unicellular analysis, capable of quantitatively assessing more than 40 parameters per individual cell while maintaining the throughput necessary for scrutinizing millions of cells within biological samples (Spitzer and Nolan, 2016). Numerous research groups have explored new element mass labels and highly sensitive mass labels. Winnik’s research group introduced the mass tag for MC immunoassays in single-cell analysis, which was dependent on tantalum oxide nanoparticles (NPs). It realized the measurement of trace protein markers within individual cells (Zhang et al., 2020). In this work, three conjugates of Ab-NPs were created, including TaO_2_-PEG_2k_-goat antimouse, TaO_2_-PEG_2k_-CD25, and TaO_2_-PEG_2k_-CD196, and assessed their performance in MC immunoassays. The results demonstrated that a secondary Ab-NPs conjugate (TaO_2_-PEG_2k_-goat antimouse) successfully detected numerous CD20 biomarkers on Ramos cells, while two primary Ab-NPs conjugates (TaO_2_-PEG_2k_-CD25, TaO_2_-PEG_2k_-CD196) effectively detected low-abundance CD25 and CD196 biomarkers on T_reg_ cells and Th17-like cells in human PBMCs. Good et al. (Good et al., 2019) developed a fluorescent immunolabel for tracking and monitoring the proliferation process of human T cells *in vitro*. By introducing 23 fluorescently labeled molecules, this method can efficiently identify and quantify protein molecules regulated by division state or division time, enabling real-time monitoring during the differentiation of specific protein groups in individual cells. This cell fate tracing method across division states and time holds broad applicability for guiding cell differentiation, proving particularly significant for the advancement of cell-based cancer immunotherapy in clinical settings. In another study, Tanaka et al. (Tanaka et al., 2022), featuring a histidine label, using single-cell inductively coupled plasma mass spectrometry (SC-ICP-MS), have devised a rapid and precise quantification technique for measuring the amount of recombinant protein expressed in *E. coli* (*E. coli*). They introduced a plasmid vector containing either the enhanced green fluorescent protein (EGFP) or red fluorescent protein (mCherry) gene, both equipped with a histidine label, into *E. coli* via transformation. Afterward, the transformed *E. coli* underwent treatment with either nickel (Ni) chloride or cobalt (Co) chloride to attach Ni or Co ions to the histidine tag. Subsequently, SC-ICP-MS was employed to evaluate the quantities of EGFP or mCherry protein by leveraging the information obtained from Ni or Co binding to the histidine label. The SC-ICP-MS technique proves valuable in accurately quantifying soluble recombinant proteins in *E. coli*, eliminating the need for extraction and purification.

**FIGURE 12:**
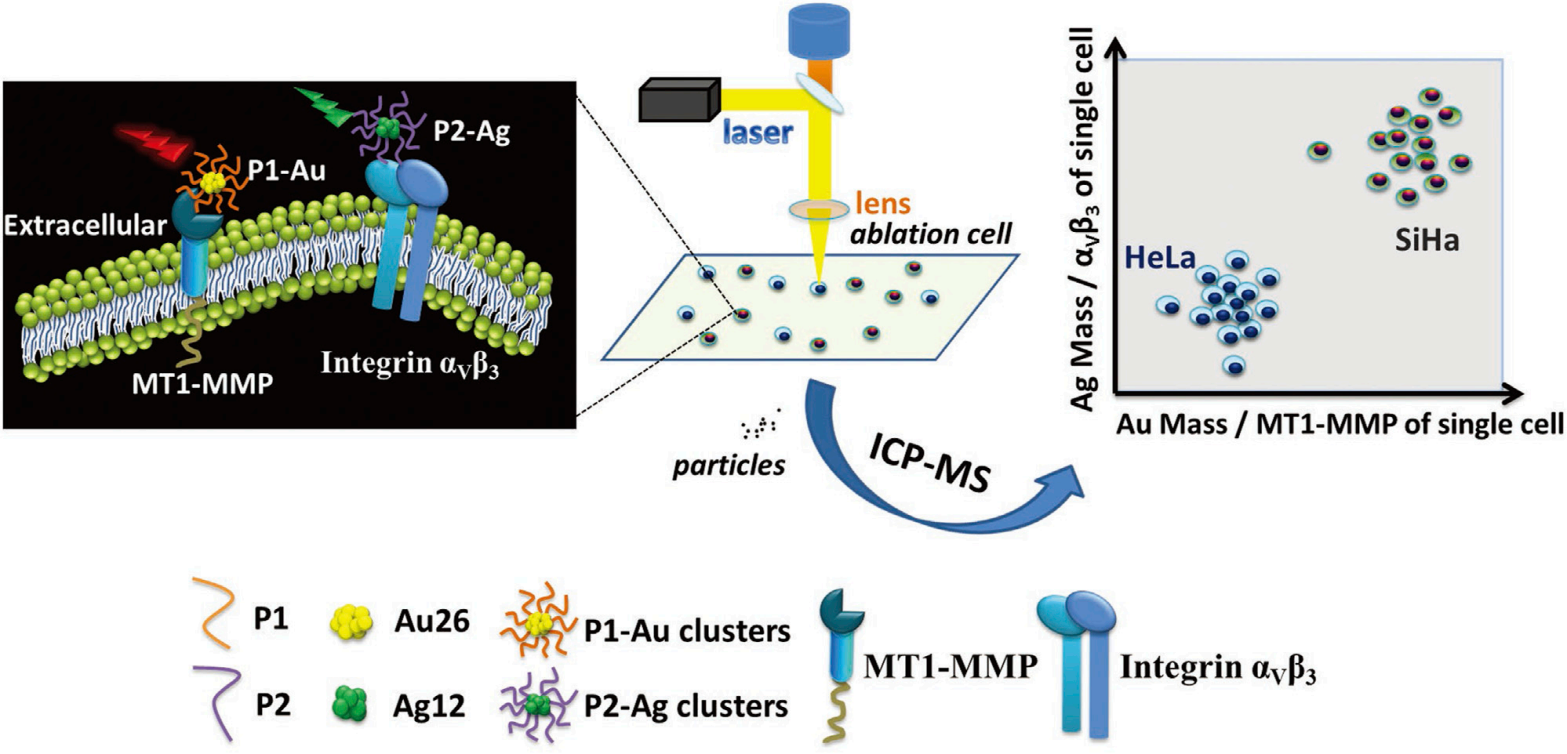
The quantitative evaluation of the invasion capability of individual SiHa and HeLa cells using LA-ICP-MS with the aid of metal clusters. Adapted with permission from Zhang et al. (2018d). *Small* 14, 1,703,684 (Zhang et al., 2018d). Copyright 2018, Wiley Online Library.

Laser ablation (LA) serves as a sampling introduction mechanism or microsampling probe, mainly utilized for sampling tissue sections or cellular monolayers into ICP-MS (Becker et al., 2007; Becker et al., 2010; Wang et al., 2014). LA-ICP-MS offers straightforward sample preparation and highly sensitive multi-elemental examination, providing a low μm-level lateral resolution for analyzing single cells or imaging subcellular organelles (Mueller et al., 2014; Pozebon et al., 2017). Herrmann and others (Herrmann et al., 2017) proposed a straightforward metal labeling protocol to identify and image individual cells using LA-ICP-MS. This method was applied to Swiss albino mouse fibroblast cells (NIH-3T3) and human lung epithelial cells (A549). An iridium intercalation agent was employed for cell nucleus staining, while the entire cell was stained with a maleimide-mono-amideDOTA (mDOTA) complex containing lanthanide (III) ions. This approach enabled the quantification of metal stain quantities ranging from 2.34 to 9.81 fmol per cell, facilitating the direct recognition and visualization of individual cells and cellular components (i.e., DNA) through elemental microscopy, eliminating the need for bright-field sample images. Zhang et al. (Zhang et al., 2018d) present an approach for quantitatively analyzing multiple proteins in various invasive tumor cell lines in individual cells using LA-ICP-MS, aided by metal clusters (Figure 12). To differentiate between tumor cells with varying invasive capabilities, two peptides with different fluorescent characteristics were developed: green-emissive Ag_12_ clusters and red-emissive Au_26_ clusters. These peptides enable the simultaneous assessment of MT1-MMP and integrin α_V_β_3_ expression levels within identical individual tumor cells. Au_26_ clusters displayed a distinct affinity for MT1-MMP, while Ag_12_ clusters exhibited a particular attraction to integrin α_V_β_3_. By using the unique fluorescent characteristics and metal components of metal clusters, this approach enabled the concurrent and quantitative assessment of MT1-MMP and integrin α_V_β_3_ levels in both highly invasive SiHa cells and less invasive HeLa cells via LA-ICP-MS. The capability of quantitatively detecting multiple invasive proteins within the same cell is of significant value for precise invasive tumor diagnosis and monitoring tumor invasiveness.

Compared with other pretreatment technologies, microfluidics is characterized by its extensive integration and microscale channels, which are very conducive to accurate cell operation (Dusny and Grunberger, 2020). Hu and others (Chen et al., 2022) proposed a microchip featuring negative magnetophoresis-based focusing, incorporating dual Y-type channels and dual divisions. This complicated microchip, which operates without the need for an organic phase, was seamlessly integrated with ICP-MS to enable unicellular analysis with high throughput. The chaotic assembly of cells was meticulously tailored to a single-cell flow through the synergistic action of magnetic repulsion and inertial lift forces, facilitating their seamless integration with ICP-MS. This integration enabled the real-time analysis of zinc content in individual MCF-7 cells subjected to treatments with either Zn^2+^ or ZnO NPs. The cellular analysis findings revealed that the absorption of ZnO NPs was lower compared to that of Zn^2+^, and greater dissimilarity was observed among the cells taking up ZnO NPs. In the conditions that were fine-tuned, the developed approach achieved a detection throughput of 1,390 cells/min. This approach offers several advantages, including straightforward sample preparation, reduced sample usage, a small dead bulk, and without an organic phase.

## Conclusion

The ongoing progress in cell biology has led to growing demands for enhanced analytical ability in single-cell MS methodologies. On the one hand, with the deepening research of single-cell multi-omics studies, it has become an urgent need to develop novel MS methods with higher throughput and wider molecular coverage. On the other hand, the need to accurately quantify low-abundance compounds in individual cells greatly demands heightened sensitivity, precision, and specificity in single-cell analytical methods and instruments. Facing the ever-growing demands for single-cell MS analysis, existing ionization methods like nano-ESI, LDI, SIMS, and ICP are undergoing constant refinement and enhancement. Currently, the evolution of these techniques aligns with the following trends:

(1) *In situ* non-destructive single-cell measurement. Currently, most single-cell ionization techniques involve the destruction of the cell, limiting the real-time analysis of intracellular components during cell proliferation or drug treatment. By creating minimally destructive online sampling techniques and highly effective ionization methods, future development of online, non-destructive, and authentic single-cell analysis is possible. This would allow reliable measurement of trace substances within single cells in their native environments.
(2) Conducting metabolomics analysis in single cells at high throughput. Although analytical throughput in unicellular metabolomics has improved significantly with MC and MALDI-MS, achieving exceptional throughput analysis in individual cells remains a formidable challenge across multiple domains. For example, automated analysis of thousands of tissue cells from clinical samples. Currently, research teams have developed novel methods combining ionization techniques with microfluidics to achieve simultaneous, exceptional throughput analysis of different cell types (Yao et al., 2019; Xu et al., 2020; Xu et al., 2021; Feng et al., 2022). Looking ahead, we may attain automation, miniaturization, and high-throughput rapid analysis of individual cells by integrating MS ionization techniques with microfluidics, flow cytometry, and other cutting-edge technologies.
(3) Single-cell MS for precise quantification research. Simple qualitative or semi-quantitative analysis can no longer meet the increasingly profound research demands of cellular research. Currently, through methods like isotope dilution MS, researchers have made preliminary achievements in the accurate quantification analysis of specific compounds within single cells. To achieve more precise and comprehensive quantification of various biomolecules within single cells, there is a prospect of developing standard artificial cells containing certain compounds within single cells. By combining different single-cell MS methodologies, high-accuracy, high-stability, and high-sensitivity MS quantification platforms are expected to be established in the future, enabling precise quantification and traceability studies of different components within single cells.
(4) Single-cell multi-omics research. With the continuous improvement in MS techniques, accurate measurement of different specific components within single cells has been achieved, such as nucleic acids, proteins, lipids, small molecule metabolites, metal elements, etc., leading to the emergence of the trend of single-cell multi-omics research. In the future, there will be a convergence and deepening of research efforts across various omics disciplines. By integrating various single-cell MS analysis methodologies, a broader spectrum of qualitative and quantitative insights into multi-omics at the unicellular level can be acquired. Ultimately, this will effectively serve significant scientific domains, including early clinical diagnosis, drug development, and ecological environment protection.
